# Better Resolution of High-Spin Cobalt Hyperfine at Low Frequency: Co-Doped Ba(Zn_1/3_Ta_2/3_)O_3_ as a Model Complex

**DOI:** 10.3390/ijms19113532

**Published:** 2018-11-09

**Authors:** William E. Antholine, Shengke Zhang, Justin Gonzales, Nathan Newman

**Affiliations:** 1 Department of Biophysics, Medical College of Wisconsin, Milwaukee, WI 53226, USA; 2 Materials Program, Arizona State University, Tempe, AZ 85287, USA; szhang76@asu.edu (S.Z.); jmgonz39@asu.edu (J.G.); nathan.newman@asu.edu (N.N.)

**Keywords:** electron paramagnetic resonance, EPR, multifrequency EPR, high-spin cobalt complex, resolution of ***A***-mid

## Abstract

Low-frequency electron paramagnetic resonance (EPR) is used to extract the EPR parameter ***A***-mid and support the approximate X-band value of ***g***-mid for Ba(Co_y_Zn_1/3−y_Ta_2/3_)O_3_. Although the cobalt hyperfine structure for the |±1/2〉 state is often unresolved at X-band or S-band, it is resolved in measurements on this compound. This allows for detailed analysis of the molecular orbital for the |±1/2〉 state, which is often the ground state. Moreover, this work shows that the EPR parameters for Co substituted into Zn compounds give important insight into the properties of zinc binding sites.

## 1. Introduction

The electron paramagnetic resonance (EPR) parameters of high-spin cobalt(II) (Co^2+^) complexes in small molecules and enzymes consist of ***g***-values, zero field splitting parameters D and E/D, and sometimes resolution of the Co hyperfine for the | ± 3/2〉 doublet; most often, they do not include resolution of the Co hyperfine for the | ± 1/2〉 doublet [1,2,3]. Copper hyperfine and superhyperfine lines, particularly the hyperfine lines about ***g***-parallel, are better resolved at low frequencies due to less ***g***- and ***A***-strain [4,5,6,7]. To the best of our knowledge, this is the first paper to show that the hyperfine lines for high-spin Co for the Co site in Co-doped barium zinc tantalite (BZT) (i.e., Ba(Zn_1/3_Ta_2/3_)O_3_) are resolved for the | ± 1/2〉 doublet at low frequencies. The determination of Co ***A***-mid provides an EPR parameter that is more sensitive to adduct formation and the electron density distribution in the | ± 1/2〉 state. It is anticipated that recording hyperfine values due to the better resolution at low frequency along with a more accurate determination of the ***g_eff_***-mid value would lead to the classification of types of cobalt sites and to identification of nitrogen, oxygen, and sulfur donor atoms, as is done for cupric sites [5]. Then, ***A***-mid for Co could be used to characterize zinc (Zn) sites, where Co is substituted for Zn.

## 2. Results and Discussion

### 2.1. No Resolvable Hyperfine Structure at X-Band

**Figure 1:**
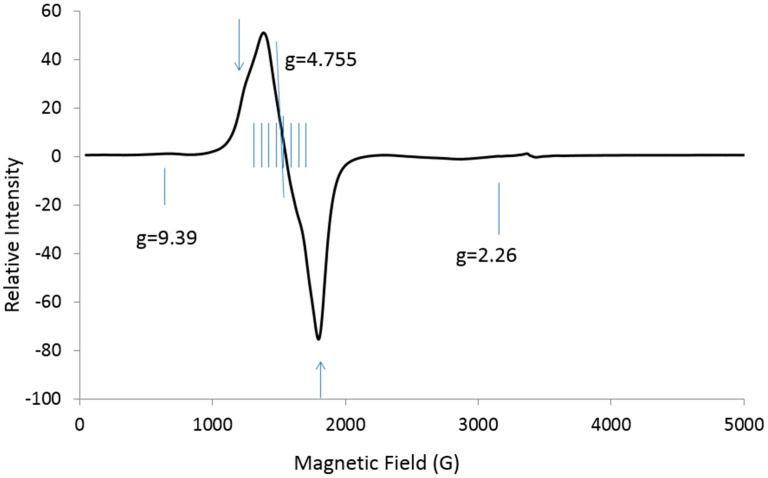
X-band (9.488 GHz) spectrum at 12 K for Co-doped barium zinc tantalate (BZT); eight short vertical lines for expected but unresolved Co hyperfine lines; vertical lines with arrows may depict exchange-narrowed and/or dipolar-broadened lines for the interaction of the nearest neighbors. Weak lines at ***g*** = [9.39 and 2.26] are not assigned.

EPR spectra were obtained for Ba[(Zn_1−y_Co_y_)_1/3_Ta_2/3_]O_3_, where y is 0.03. The X-band spectrum gives a central ***g_eff_*** value of 4.76 and possibly exchange-narrowed and/or dipolar-broadened lines for the interaction of the nearest neighbors, but the Co hyperfine structure is unresolved (Figure 1) [8].

### 2.2. Cobalt Hyperfine Lines Resolved at Low Frequencies: S-Band and L-Band

**Figure 2:**
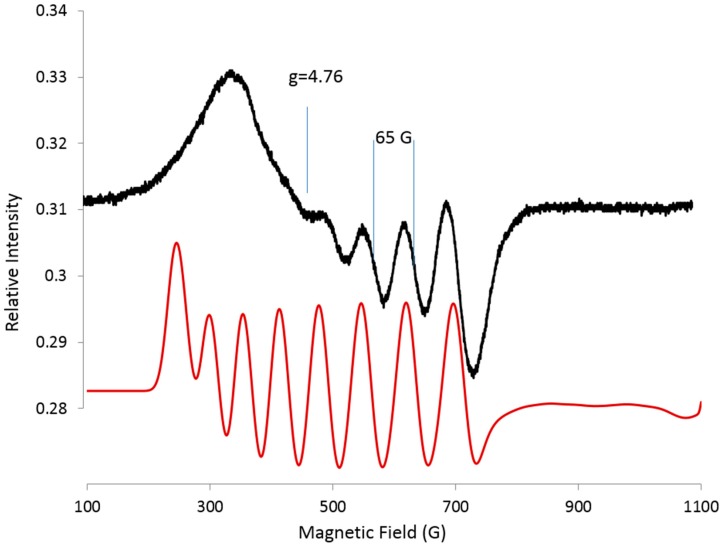
S-band (3.216 GHz) spectrum (black) for Co-doped BZT at 17 K. Exp: 28 dB, 5 G mod., time constant 0.1285 s. Simulation (red): EasySpin, least squares, simplex, ***g_eff_*** = [4.83, 4.56, 2.14], ***A*** = [432, 402, 130 (fixed) MHz], ***HStrain*** = [200, 200, 200 MHz].

The S-band spectrum (3.216 GHz) has four of eight resolved Co hyperfine lines, for which a ***g_eff_***-mid value of 4.76 and an ***A***-mid of 65 G (432 MHz) are readily apparent (Figure 2). A simulation using EasySpin and a least squares fitting routine gives ***g_eff_*** values [4.83, 4.56, 2.14] and ***A*** values [432, 402, 130 MHz] (Figure 2). The simulation is consistent with, but not proof of, the parameters for the experimental spectrum, because other parameters such as line width variation, Euler angles, etc., are not included and the simulation may not be unique. The number of variables is underdetermined for three multifrequency spectra. Simulations suggest that the structure is slightly rhombic, but the ***g_eff_*** value of 4.76 confirms that E/D falls close to the tetragonal value. Nevertheless, clear values for ***g_eff_***-mid and ***A***-mid are obtained. A ***g_eff_*** value of 4.8 suggests that the ***g***-mid and ***A***-mid are for the | ± 1/2〉 state from the rhombogram [9].

**Figure 3:**
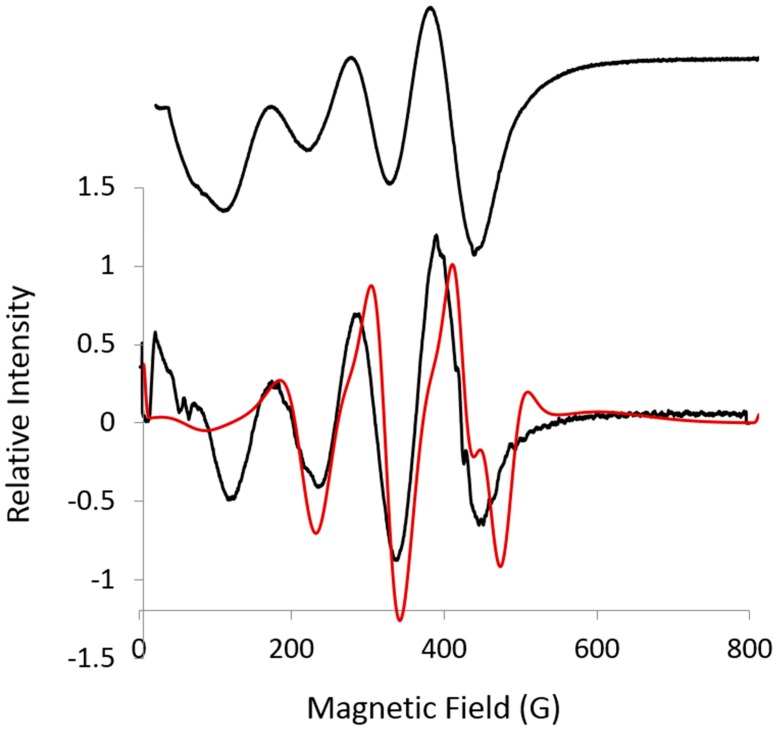
L-band (1.362 GHz) spectrum (black, top) at 16.7 K. Exp: 28 dB, 5 G mod., time constant 0.128 s; second harmonic (black, bottom) 1% Bessel function using Sumspc, see Section 3.2 for details; Simulation (red), EasySpin, least squares, Monte Carlo: ***g*** = [5.04, 4.01, 2.14 (fixed)], ***A*** = [472, 393, 130 (fixed) MHz].

The L-band spectrum is well resolved but complicated (Figure 3). None of the splittings between the resolved lines directly correspond to the 65 G for ***A***-mid obtained at S-band, presumably from the overlap of lines. It is difficult to determine whether the resolved lines are S-shaped, as would be obtained for ***g_eff_***-mid and ***A***-mid, or hills and valleys, as would be obtained for the low- and high-field EPR parameters. The second harmonic of the L-band spectrum was measured to accurately characterize the line shape (Figure 3), as it does very well for sharp lines. Starting with parameters from S-band, a simulation (red spectrum) was obtained using EasySpin with least squares fitting by Monte Carlo (Figure 3). The EPR parameters obtained are ***g_eff_*** = [5.04, 4.01, 2.14], where 2.14 is arbitrarily fixed, i.e., set without resolved or even unresolved lines, and ***A*** = [472, 393, 130 (fixed) MHz]. ***A***-max from the simulation equals 67 G and ***A***-mid equals 67 G; these are in good agreement with the S-band value of 65 G. The simulation is also consistent with the experimental parameters (Table 1). It is noted that the simulations come with a warning that there are looping transitions and possible discontinuities at the ends of the spectrum. Looping transitions occur when S = 3/2 and pairs of non-crossing levels vary nonlinearly with the magnetic field [10]. A single pair of energy levels is in resonance before and after a crossing or near-crossing [11]. However, the agreement with the experimental spectrum is evidence that the simulated parameters are sensitive to ***g_eff_*** and ***A*** values.

### 2.3. Summary

***A***-mid and ***g_eff_***-mid from low-frequency spectra for Ba[(Zn_1−y_Co_y_)_1/3_Ta_2/3_]O_3_, where y is 0.03, were determined from the experimental S-band spectrum and from simulations of S-band and L-band EPR spectra. It was estimated that E/D from a rhombogram is less than or equal to 0.1, indicating that the crystal field is tetragonal, almost octahedral. This study shows that the Co hyperfine value can be obtained for the | ± 1/2〉 ground state for high-spin Co^2+^ complexes. This is the first Co site for which we have resolved both S-band and L-band hyperfine spectra. Thus, these spectra for Co-doped BZT serve as a model for Co complexes where the Co hyperfine is resolved at L-band but not S-band, as is usually found in other complexes [12,13]. It is suggested that EPR values for ***A***-mid will be a more sensitive parameter for determining the coordination of and differences in the coordination of high-spin Co complexes. Zn sites outnumber cobalt sites and other metal sites in metalloenzymes [2]. Substitution of cobalt for zinc provides a paramagnetic site using the ligands for a non-paramagnetic zinc site or using the same ligands plus one or two ligands by, for example, expanding a four-coordinate tetrahedral site to a five- or six-coordinate site [2]. Perhaps the most important use of cobalt EPR may be as a substitute to probe zinc sites.

## 3. Materials and Methods

The methods to obtain the microwave ceramic, Co-doped BZT (Co-doped Ba(Zn_1/3_Ta_2/3_)O_3_), are given in reference [8].

### 3.1. Molecular Structure

Co^2+^ ions are substituted for Zn^2+^ in Ba(Zn_1/3_Ta_2/3_)O_3_ [8]. The Co^2+^ ions are in a slightly distorted octahedral crystal field, and the ground state has symmetry T1g.

### 3.2. EPR Spectrometers

Data were obtained from a low-frequency spectrometer station assembled at the National Biomedical EPR Center at the Medical College of Wisconsin (Milwaukee, WI, USA). The station incorporates an in-house-built L-band (1–2 GHz) bridge, Varian V-7200 Electromagnet, Varian V-7700 Magnet Power Supply, and Bruker BH-15 Magnetic Field Controller. The 100 KHz field modulation and signal phase-sensitive detection were provided by a Varian E-109 System EPR console. EPR signals from the phase-sensitive detector were recorded on a PC with Windows 7 running a custom LabVIEW program. The program also controlled the BH-15 Field Controller and performed multiple-scan signal averaging, when needed.

The L-band bridge in the spectrometer utilizes a low-phase-noise, mechanically and electronically tunable fundamental transistor oscillator capable of 50 mW power output to the sample resonator port at 0 dB main power attenuator setting. A loop-gap resonator was used to collect samples [6,7]. The oscillator microwave frequency was locked to the sample resonator frequency by a 70 KHz automatic frequency control system in the bridge, operating through the electronic tuning port of the oscillator. A low-noise amplifier in the microwave signal receiver prior to signal mixing improved the overall bridge sensitivity.

L-band simulations were completed assuming that S = 1/2 using an online version of EasySpin [14]. Starting with parameters from S-band, ***A***-max, ***A***-mid, ***g***-max, and ***g***-mid were varied over a weekend using the Monte Carlo option. The second harmonic was obtained using the SUMSPC program developed by J. Ratke at the National Biomedical EPR Center at the Medical College of Wisconsin (Milwaukee, WI, USA). Sumspec is available at no cost from the National Biomedical EPR Center (Milwaukee, WI, USA).

S-band spectra (3.2 GHz) were acquired using a loop-gap resonator, which is one of the spectrometers housed at the National Biomedical EPR Center at the Medical College of Wisconsin (Milwaukee, WI, USA) [6,7]. The hyperfine constant for Co at S-band was obtained by taking the value from the spectrum assuming an S-shaped line (Figure 2). The g value was obtained by taking the center of the eight-line pattern of which four lines are resolved (Figure 2). The ***g_eff_*** values were set at ***g*** = [4.76, 4.76, 2.14] and ***A*** = [432, 432, 100]. The first *A*_min_ and *A*_mid_ were fit using the Nelder/Med simplex least squares routine from EasySpin; then, *g*_min_ and *g*_mid_ were varied, assuming a spin of S = 1/2.

X-band spectra were obtained at Marquette University (Milwaukee, WI, USA) using an updated EMX spectrometer and a Bruker cryogen-free system. The only parameter taken from the X-band spectrum was the *g* value at the center of the S-shaped line, ***g*** = 4.76 (Figure 1), as calculated from the resonant frequency from the frequency counter and the magnetic field at the center of this line.

## Figures and Tables

**Table 1:** Electron paramagnetic resonance ***A***-mid values and ***g_eff_*** for Co-doped BZT from spectra and simulations.

	*g*-max	*g*-mid	*A*-max	*A*-mid
X-band (9.488 GHz, exp)	------	4.76	------	------
S-band (3.216 GHz, exp)	------	4.76	------	64.9 G
S-band (3.216 GHz, sim)	4.83	4.56	63.8 G	63.0 G
L-band (1.362 GHz, sim)	5.04	4.01	66.9 G	67.0 G

## Abbreviations

BZT	barium zinc tantalate
Co	cobalt
EPR	electron paramagnetic resonance
Zn	zinc
